# From Awareness to Action: Advancing Counselor Education Policy to Improve Early Detection of Eating Disorders Through Embodied Reflection

**DOI:** 10.3390/bs16091618

**Published:** 2026-09-10

**Authors:** Rebecca E. Taylor, Sarah Jarvie

**Affiliations:** School of Counseling, Colorado Christian University, Lakewood, CO 80226, USA

**Keywords:** eating disorders, counselor education, early detection, embodied self-awareness, body image, weight stigma, clinical training

## Abstract

Eating disorders are associated with substantial morbidity and mortality yet remain underidentified in general mental health practice, delaying intervention and worsening outcomes. Counselor preparation often provides limited eating disorder content within broader psychopathology coursework, leaving trainees underprepared to recognize subtle, culturally diverse, and nonstereotypical presentations. This conceptual paper draws on scholarship in counselor education, body image, embodied self-awareness, and eating disorder training to propose a four-domain framework intended to strengthen counselor preparation for eating disorder recognition and clinical response. The framework includes foundational knowledge of eating disorder diagnosis, medical and nutritional risk, levels of care, and multidisciplinary treatment; reflective body-related self-awareness concerning clinicians’ beliefs about food, weight, health, exercise, appearance, and culture; embodied and creative practices that strengthen awareness of bodily and relational responses; and clinical translation through direct assessment, supervision, consultation, and referral. A seven-session graduate course illustrates how the framework can be implemented while protecting students from coerced personal disclosure. Within the U.S. counselor education and mental health training context, policy recommendations address curriculum infusion, weight stigma, countertransference, competency assessment, and basic detection and referral preparations. Integrating body-related reflection with diagnostic knowledge may offer a promising approach for helping clinicians recognize when eating disorder and body image concerns warrant further inquiry, reduce stereotype-based omissions, and respond more deliberately across diverse clinical settings; however, whether embodied training improves detection outcomes remains an empirical question.

## 1. Advocating for Embodied Training

Mental health training programs have long recognized that the person of the clinician matters. Within counselor education, professional formation includes attention to ethical practice, professional identity, cultural responsiveness, values, supervision, and the reflective use of self. The Multicultural and Social Justice Counseling Competencies further emphasize counselor self-awareness as foundational to culturally responsive practice, calling counselors to examine their assumptions, values, beliefs, biases, social identities, experiences of privilege and marginalization, and participation in systems of power and oppression (Ratts et al., 2016). In eating disorder training specifically, recent counselor education scholarship has similarly called for increased trainee self-awareness, culturally responsive conceptualization, and interdisciplinary collaboration as essential components of counselor preparation (Irvine & Labarta, 2024). Across these frameworks, trainees are asked to consider how their identities, values, histories, and worldviews shape the care they provide.

Despite this emphasis on self-awareness, the body often remains unnamed or only indirectly addressed. Trainees may examine race, ethnicity, religion, gender, family of origin, trauma history, values, and power while receiving little structured opportunity to examine their own relationships with body image, food, health, weight, exercise, appearance, sexuality, aging, disability, fatigue, hunger, fullness, and bodily discomfort. This omission is significant because the clinician’s body is not separate from clinical perception, assessment, or relational presence. Embodied self-awareness has been described as a crucial but often neglected dimension of counselor education, involving the capacity to attend to internal bodily states, interoceptive and proprioceptive cues, emotional responses, and relational attunement in the therapeutic encounter (Rodríguez & Wilkinson, 2026). Yet, in most training contexts, embodied awareness is not consistently connected to eating disorder prevention, early detection, body image assessment, or the examination of clinician bias toward different body sizes, eating patterns, health narratives, and appearances.

This gap has direct implications for eating disorder identification and care. Eating disorders and body image distress are often hidden, minimized, normalized, or misidentified, and many individuals do not receive timely or adequate treatment despite the seriousness of these conditions and the demonstrated importance of early intervention (Kazdin et al., 2017; Mehler & Andersen, 2022). Clients may initially present with anxiety, depression, trauma symptoms, perfectionism, compulsive exercise, gastrointestinal concerns, menstrual irregularities, shame, relational distress, spiritual distress, or concerns about control rather than explicitly naming disordered eating or body image suffering. When clinicians are not trained to ask informed, culturally responsive, and body-aware questions, they may unintentionally “rule out” eating disorder concerns too quickly, particularly when clients do not fit stereotypical assumptions about who struggles with eating disorders.

Recent scholarship has begun to address portions of this problem. Irvine and Labarta (2024) proposed the 3 Cs of eating disorder education and training: cultivating trainee self-awareness, capturing contextual and sociocultural factors, and collaborating with interdisciplinary eating disorder professionals. Their framework moves counselor education toward a more intentional and culturally responsive model of eating disorder preparation. Labarta et al. (2023) further demonstrated the urgency of this need, finding that clinicians identified lack of graduate-level education and specialized training as a major challenge in treating eating disorders and recommending increased attention to countertransference, culturally responsive care, and specialized coursework. Building on this work, the present article argues that eating disorder training must move one step further by explicitly integrating body-based self-awareness into counselor formation. In this model, trainees are not only asked to reflect generally on beliefs and biases, but to examine how their embodied histories, body-related values, assumptions about health and weight, food rules, appearance ideals, and discomfort with bodily vulnerability may shape what they notice, avoid, ask, normalize, pathologize, or miss in clinical practice.

The purpose of this article is to move from awareness to action. Rather than merely encouraging clinicians to “be self-aware,” this article argues that mental health training programs should intentionally teach, practice, supervise, and assess embodied, reflective, body-based self-awareness as part of eating disorder awareness and prevention. Specifically, body-based counselor formation should be understood as a values-based and culturally responsive practice that helps clinicians identify their own body-related assumptions and biases while increasing their capacity to maintain possible eating disorder and body image concerns within the differential when further inquiry is warranted, rather than prematurely excluding them on the basis of stereotypical assumptions. A four-domain framework is proposed, followed by a course-based example, policy recommendations, ethical safeguards, and a hypothesis-driven research agenda.

## 2. Method of Conceptual Development

This article was developed through a focused review of scholarship relevant to eating disorder training, counselor self-awareness, body image, embodied awareness, and clinical education. Sources included peer-reviewed conceptual and empirical literature, professional training standards, and relevant accreditation and ethical guidance, with emphasis on contemporary scholarship while retaining foundational sources necessary to define key constructs. Literature was selected based on its relevance to counselor preparation, eating disorder recognition, body-related bias, embodied awareness, and clinical translation; literature focused primarily on specialized eating disorder treatment outcomes was generally outside the scope of the review. The four domains were not derived through a formal systematic or qualitative analytic procedure. Rather, they represent the authors’ conceptual organization of recurring priorities across these bodies of scholarship, with embodied and creative practice proposed as an extension intended to connect existing self-awareness recommendations more explicitly to bodily experience and clinical application.

## 3. Self-Awareness Is Already a Professional Expectation

The argument for embodied and reflective body-based learning does not require abandoning existing professional standards. Rather, it extends ethical, multicultural, and developmental commitments already embedded within mental health training. Because the regulatory and accreditation analysis in this article draws primarily on U.S.-based professional standards, the discussion that follows is intentionally situated within the United States. CACREP, ACA, APA-CoA, and ACGME standards are used to illustrate how embodied and reflective learning may align with existing expectations for professional preparation across U.S. mental health disciplines. Although the broader conceptual framework may have relevance internationally, accreditation structures, professional standards, and educational requirements vary across countries. Accordingly, application of the framework outside the United States would require adaptation to the relevant local training, professional, and regulatory context.

Within U.S. counselor education, the 2024 Council for Accreditation of Counseling and Related Educational Programs (CACREP) Standards explicitly require instruction in “self-care, self-awareness, and self-evaluation strategies for ethical and effective practice” and address the influence of cultural identities, attitudes, values, beliefs, stereotypes, discrimination, power, privilege, marginalization, and health disparities on counselors and clients (Council for Accreditation of Counseling and Related Educational Programs [CACREP], 2024). CACREP also identifies culturally responsive relationships, critical thinking, clinical judgment, and adaptation to clients’ cultures, contexts, abilities, and preferences as foundational elements of counselor preparation.

These accreditation expectations are reinforced by counseling’s ethical and multicultural frameworks. The American Counseling Association (ACA) Code of Ethics requires counselors to recognize and avoid imposing their own values, attitudes, beliefs, and behaviors and to seek additional training when their values may conflict with clients’ goals or operate in discriminatory ways (American Counseling Association [ACA], 2014, Standard A.4.b). The code also requires counselor educators to infuse multicultural and diversity competence throughout training and supervision and to prepare students in the awareness, knowledge, and skills necessary for multicultural practice (American Counseling Association [ACA], 2014, Standard F.11.c). Similarly, the Multicultural and Social Justice Counseling Competencies position counselor self-awareness as a foundational domain, asking counselors to examine their cultural values, beliefs, biases, assumptions, social identities, and experiences of power, privilege, and marginalization (Ratts et al., 2016).

Body-based reflection is therefore not peripheral to counselor identity or multicultural competence. For example, beliefs about food, weight, health, attractiveness, fitness, disability, and aging, just to name a few. These beliefs may also function as values that clinicians unintentionally impose. For example, a counselor may assume that weight loss is inherently desirable, that thinness signifies health, that exercise is always adaptive, that certain foods reflect discipline or morality, or that eating disorder severity should be visible in a client’s body. When such assumptions remain unexamined, they may influence assessment questions, diagnostic impressions, clinical language, treatment goals, referrals, and the therapeutic relationship. Counselor self-awareness must therefore include not only what clinicians believe about cultural difference in the abstract, but also how cultural values and biases become attached to bodies.

Recent research demonstrates why this extension is necessary. Mental health training programs may provide insufficient preparation related to weight bias, body size, and clinical work with larger-bodied clients, leaving culturally reinforced associations among weight, health, morality, and personal responsibility insufficiently examined (Philip et al., 2025). The consequences are clinically meaningful: provider judgments may be affected by client body size, and restrictive eating disorder symptoms may be perceived as less severe in higher-weight clients than in thinner clients presenting with comparable symptoms (Philip et al., 2025). Counselor education scholars have similarly warned that unexamined anti-fat bias places larger-bodied clients at risk of additional harm within counseling relationships and have called for trainees to examine prejudice related to body size as a matter of multicultural competence and social justice (Kerl-McClain et al., 2022).

Within U.S. health service psychology, comparable expectations are also evident. The American Psychological Association (APA) Commission on Accreditation identifies profession-wide competencies in ethical and legal standards, individual and cultural diversity, professional values, attitudes, and behaviors, communication and interpersonal skills, assessment, intervention, supervision, consultation, and interprofessional skills (American Psychological Association, Commission on Accreditation [APA-CoA], 2024). The accompanying implementing regulations require trainees to understand how their own personal and cultural histories, attitudes, and biases may affect how they understand and interact with people who differ from themselves (American Psychological Association, Commission on Accreditation [APA-CoA], 2024). APA’s multicultural guidelines likewise conceptualize psychologists as cultural beings whose identities, assumptions, and social locations shape professional perception and practice (American Psychological Association [APA], 2017). Body-related assumptions fit directly within this expectation because they may shape what clinicians ask, what they normalize, how they interpret symptoms, whose distress they recognize, and whose concerns they minimize.

U.S. psychiatry training offers another relevant comparison. The Accreditation Council for Graduate Medical Education (ACGME) Psychiatry Milestones explicitly identify the use of clinician and patient emotional responses as diagnostic information within psychiatric formulation and differential diagnosis (Accreditation Council for Graduate Medical Education [ACGME], 2020). Across developmental levels, residents are expected to progress from recognizing that clinicians have emotional responses to patients, to recognizing the potential diagnostic value of those responses, to integrating them into formulation and diagnosis. This is conceptually important because it recognizes the clinician’s internal experience as potentially relevant to clinical understanding rather than merely incidental.

Embodied responses deserve similarly disciplined attention. For example, a trainee’s tightening, feelings of numbness or shame may reflect countertransference, cultural conditioning, perceived difference, implicit bias, or other aspects of the clinician’s own experience. These reactions should not be treated as inherently accurate information about the client, nor should they be acted upon uncritically. Instead, they can be noticed, questioned, contextualized, and processed through reflection and supervision. Embodied self-awareness thus involves learning to distinguish between potentially useful clinical awareness and reactions arising from the clinician’s own body history, cultural learning, fear, shame, or internalized assumptions.

Therefore, the question is not whether U.S. mental health professions value self-awareness; existing professional standards demonstrate that they do. The more precise question is whether current training sufficiently names the body as a site of values, identity, bias, countertransference, and clinical awareness. Existing standards provide a strong ethical and multicultural foundation, but they rarely operationalize how trainees should examine their relationships with food, weight, health, appearance, movement, disability, sexuality, aging, hunger, fullness, and bodily discomfort. This article proposes that this gap may be addressed through intentional embodied and reflective pedagogy that complements, rather than replaces, existing expectations for ethical practice, multicultural competence, clinical judgment, and the reflective use of self.

## 4. The Missing Body: Body Attitudes, Health Values, and Disembodiment

The body is not merely a biological object, diagnostic variable, or container for symptoms. It is also a site of identity, culture, memory, pleasure, shame, trauma, power, spirituality, social evaluation, and belonging. Embodiment scholarship describes the body as the means through which individuals experience and participate in the world, rather than as a passive vessel occupied by the mind (Rodríguez & Wilkinson, 2026). Similarly, the developmental theory of embodiment emphasizes that experiences of living in the body are shaped by social conditions that either support or constrain body connection, agency, self-care, bodily desire, and freedom from objectification (Piran et al., 2023). Thus, clients do not simply possess bodies that occasionally become relevant to treatment; they encounter themselves, relationships, institutions, and cultural expectations through their embodied lives.

Clients bring these embodied experiences into counseling in both explicit and indirect ways. Some name explicit body experiences such as hatred, eating disorder behaviors, gender-related body distress or dysphoria, chronic pain, compulsive exercise, body checking, food fear, sexual trauma or medical trauma. While others communicate bodily suffering less directly through avoidance, dissociation, overcontrol, perfectionism, shame, numbness, anxiety, or relational withdrawal. Difficulty recognizing, tolerating, or interpreting internal bodily signals may be relevant to the experiences of some individuals with eating disorders. Systematic reviews and meta-analytic findings have documented associations between disordered eating and disruptions in interoceptive awareness, including difficulty recognizing and trusting hunger, fullness, emotion, and other internal physiological states (Jenkinson et al., 2018; Martin et al., 2019). These findings concern clients’ interoceptive experiences and should not be interpreted as evidence that clinicians with greater interoceptive awareness are better able to detect eating disorders. Client interoceptive disturbance and clinician embodied awareness are distinct constructs, and a direct relationship between clinician interoceptive capacity and eating disorder detection has not been established.

The relevance of clinician embodiment in the present framework is therefore different. Clinicians also bring bodies into the therapeutic encounter through their own physiological responses, personal histories, values, emotions, and culturally shaped assumptions. Some clinicians may notice bodily shifts during clinical interactions and use that awareness to support regulation and therapeutic presence, whereas others may rely more heavily on cognitive processing or feel less comfortable attending to bodily sensations (Rodríguez & Wilkinson, 2026). Still others may experience activation related to their own eating histories, body shame, trauma, experiences of weight stigma, family food rules, illness, or health-related anxiety. Within this framework, noticing such responses is proposed as a form of clinician self-monitoring rather than as a means of detecting client interoceptive disturbance.

These responses do not make clinicians inherently unsafe or unfit. They make clinicians human. However, body-related histories and emotional responses become clinically consequential when they remain outside reflective awareness. Labarta et al. (2023) emphasized that the personal nature of food, eating, health, and body image can generate countertransference even among clinicians without personal eating disorder histories. Clinicians’ beliefs about health and wellness are shaped by cultural identities, worldviews, and lived experiences and may be imposed unintentionally when trainees have not been supported in examining them. Their study therefore recommended structured self-reflection related to diet culture, weight stigma, personal triggers, and countertransference as part of eating disorder preparation (Labarta et al., 2023).

Health values are especially important because concepts such as “healthy,” “fit,” “disciplined,” or “taking care of oneself” are not always clinically or culturally neutral. A clinician who associates thinness with discipline may respond to weight loss differently from a clinician trained to assess eating behavior, rate of weight change, medical instability, and psychological distress. A clinician who moralizes food may inadvertently reinforce the shame within a client’s binge–restriction cycle. A clinician uncomfortable discussing weight may avoid necessary assessment, whereas a clinician who sees exercise only as a protective wellness behavior may fail to assess compulsive, rigid, or compensatory movement. Recent research demonstrates that weight-based assumptions can directly affect recognition: healthcare professionals and laypeople were less likely to identify atypical anorexia nervosa and were less confident in the diagnosis when restrictive symptoms were presented in a higher-weight body rather than a conventionally thin body (García Moreno et al., 2025).

Body-related bias also contributes to the persistence of a narrow eating disorder stereotype centered on young, thin, affluent, cisgender White women. When this stereotype organizes clinical attention, counselors may overlook risk among men and boys, athletes, transgender and gender-diverse clients, people in larger bodies, older adults, and individuals experiencing poverty or food insecurity. Research has documented disparities in eating disorder recognition and treatment according to weight status, race and ethnicity, socioeconomic background, and sex (Sonneville & Lipson, 2018). Clinically significant restrictive eating disorders also occur among individuals at higher weights (Harrop et al., 2021), while transgender and gender-diverse students demonstrate elevated and heterogeneous eating disorder risk (Simone et al., 2022). Eating disorders among boys and young men remain an important area of concern (Nagata et al., 2020), as does disordered eating among athletes (Mancine et al., 2020). Food insecurity has also been meaningfully associated with binge eating (Abene et al., 2023). (Abene et al., 2023; Ghazzawi et al., 2024; Harrop et al., 2021; Nagata et al., 2020; Simone et al., 2022; Sonneville & Lipson, 2018). Eating disorders also occur in later life, although older adults remain underrepresented in research and may not be recognized within assessment and treatment models developed primarily for adolescents and younger adults (Mulchandani et al., 2021).

The field therefore needs language that differentiates several related but distinct constructs. Body-related self-awareness refers to the clinician’s reflective capacity to identify and examine personal histories, beliefs, values, emotions, biases, and cultural narratives related to bodies, food, weight, appearance, health, movement, ability, sexuality, and aging and to consider how these may influence clinical attention and decision-making. Attitudinal bias refers more specifically to beliefs, stereotypes, evaluative assumptions, and expectations that may shape clinical judgment, such as equating thinness with health or larger body size with poor self-control. Embodied awareness refers to the clinician’s moment-to-moment capacity to notice sensations, affective shifts, tension, numbness, activation, posture, urges, and other physiological responses within themselves during the therapeutic encounter. Somatic countertransference refers to bodily or affective responses arising within the therapeutic relationship that may be influenced by the interaction with the client, the clinician’s own history, or both; such responses require reflection and supervision rather than being interpreted as direct information about the client. These concepts may interact, but they are not interchangeable. In particular, neither attitudinal bias nor somatic countertransference should be equated with interoception, which refers more broadly to the perception and interpretation of internal bodily signals. Together, body-related self-awareness and embodied awareness support body attunement, defined as the counselor’s capacity to notice, interpret, and remain receptive to internal bodily sensations and physiological signals while remaining grounded and present within the therapeutic relationship. In the context of eating disorder awareness and prevention, body attunement also involves remaining regulated, responsive, and clinically humble when body-related material emerges. It requires neither treating the clinician’s bodily reactions as objective truth nor suppressing them as irrelevant. Instead, clinicians learn to notice these responses, examine their possible origins, and determine whether and how they may inform clinical assessment and intervention through reflection and supervision.

This approach extends embodied self-awareness scholarship by connecting moment-to-moment bodily awareness with eating disorder recognition, body-related bias, culturally responsive assessment, and the ethical responsibility to “rule in” concerns that might otherwise remain unseen. Rodríguez and Wilkinson (2026) establish the importance of embodied awareness for counselor presence, self-regulation, countertransference, and relational responsiveness; your framework applies that foundation directly to eating disorder awareness and prevention.

## 5. Why This Matters for Early Detection of Eating Disorders

Eating disorder detection requires more than memorization of diagnostic criteria. Clinicians need foundational knowledge of the Diagnostic and Statistical Manual of Mental Disorders (5th ed., text rev.; DSM-5-TR; American Psychiatric Association [APA], 2022) feeding and eating disorder diagnoses, but they also need confidence initiating assessment, recognizing possible medical and nutritional risk, consulting with interdisciplinary providers, and asking direct questions about eating patterns, restriction, binge eating, compensatory behaviors, exercise, weight history, body image, and shame. A recent rapid review concluded that limited screening practices, insufficient professional training, and barriers to help-seeking continue to contribute to low detection rates and substantial unmet treatment need (Bryant et al., 2022). This is particularly consequential because eating disorders may remain untreated for years before individuals receive specialized care, reinforcing the importance of earlier recognition and intervention (Austin et al., 2021).

Counselor preparation represents an important point of intervention. Eating disorders have historically been addressed inconsistently or only briefly within broader counselor education coursework (Levitt, 2006). More recent findings suggest that this gap persists. In Labarta et al.’s (2023) interdisciplinary sample, approximately 73% of clinicians reported that their graduate programs did not offer a course devoted exclusively to eating disorders; approximately 41% received only one to five hours of eating-disorder instruction, and nearly 27% received none. Nearly 60% identified insufficient graduate education and specialized training as a significant challenge in eating disorder treatment. The authors also highlighted clinician discomfort, countertransference, weight stigma, and the need for culturally responsive preparation. Irvine and Labarta (2024) subsequently proposed the 3 Cs (cultivating trainee self-awareness, capturing contextual and sociocultural factors, and collaborating with interdisciplinary professionals) as a framework for improving counselor preparation. The present proposal builds on these recommendations by specifying how embodied reflection and body-related self-awareness may strengthen clinicians’ willingness to maintain possible eating and body-image concerns within the differential rather than prematurely excluding them.

The distinction between screening and comprehensive assessment is important. A screening instrument may identify the need for further inquiry, but it cannot replace clinical conversation, contextual formulation, or appropriate medical and nutritional referral. Early detection requires clinicians to recognize that clients may not describe their experiences using eating-disorder terminology. They may instead present with anxiety, depression, trauma symptoms, perfectionism, gastrointestinal complaints, fatigue, dizziness, menstrual or hormonal changes, compulsive exercise, disrupted sleep, social withdrawal, rigid food practices, loss-of-control eating, or distress about health and appearance. They may also minimize symptoms because the behaviors are normalized within their families, athletic environments, religious communities, peer groups, or broader wellness culture. Bryant et al. (2022) therefore emphasized the need for improved screening and assessment practices rather than relying on obvious appearance-based indicators or client self-identification alone.

Body-image scholarship provides an additional foundation for broadening assessment. Positive body image is not merely the absence of body dissatisfaction; it is multidimensional, protective, socially situated, and influenced by body acceptance from others, cultural identity, and the ways individuals interpret body-related information (Tylka & Wood-Barcalow, 2015; Wood-Barcalow et al., 2010). These constructs can help trainees recognize that body image includes more than whether a client “likes” their appearance. Assessment may also consider body appreciation, respect, functionality, investment, avoidance, comparison, checking, shame, safety, and perceived belonging within one’s body. When trainees examine how family, media, culture, religion, medicine, relationships, and social position have shaped their own body narratives, they may become better prepared to ask how these same forces operate in clients’ lives.

Cognitive-behavioral and prevention research likewise demonstrates that body-image distress is maintained through interacting cognitions, emotions, behaviors, avoidance patterns, safety behaviors, attentional biases, and social reinforcement (Lewis-Smith et al., 2019). For trainees, reflective preparation therefore should not be confined to abstract statements such as “I value body diversity.” It should include identifying automatic body-related judgments, noticing discomfort or avoidance when food and weight arise, examining assumptions about health and discipline, and practicing compassionate, culturally responsive, and dialectical responses. Embodied reflection adds another layer by helping trainees notice tightening, urgency, comparison, judgment, numbness, or withdrawal that may occur during body-related conversations. Such reactions are not treated as facts about the client; rather, they become material for reflection, supervision, and more deliberate assessment.

Strengths-based constructs can also improve early recognition and treatment planning. Body appreciation and intuitive eating have been associated with eating-disorder recovery status, suggesting that recovery involves not only decreased pathology but also the development of more adaptive relationships with food and the body (Koller et al., 2020). These constructs should not be used to minimize active symptoms or substitute for medical evaluation. Instead, they broaden the clinician’s frame by encouraging assessment of both risk and protective factors. A clinician can ask not only whether clients restrict, binge, purge, check, or avoid, but also whether they can recognize hunger and fullness, respond flexibly to bodily needs, experience their bodies as trustworthy, or engage in movement without punishment or compensation.

Early detection is also an ethical, multicultural, and social justice issue. Eating disorders do not occur only among young, thin, affluent White women, yet this stereotype continues to organize recognition and referral. Disparities have been documented according to body size, race and ethnicity, socioeconomic background, and sex, with individuals outside the dominant stereotype less likely to be recognized or treated (Sonneville & Lipson, 2018). Intersectionality is therefore essential: eating-disorder risk and help-seeking cannot be understood apart from the combined effects of race, gender, sexuality, body size, disability, socioeconomic position, and other social identities (Burke et al., 2020).

Weight stigma is especially relevant to missed detection. Restrictive eating disorders occur in people at higher weights and can involve substantial psychological distress and medical risk even when a client does not meet low-weight expectations associated with anorexia nervosa (Harrop et al., 2021). A 2024 systematic review of 242 studies found consistent associations between experienced, anticipated, and internalized weight stigma and disordered eating cognitions and behaviors (Levinson et al., 2024). Clinicians who overvalue weight loss, equate thinness with health, or view larger bodies primarily through a weight-management lens may praise clinically concerning restriction, miss rapid weight change, or fail to investigate compulsive exercise and food fear.

Food access and economic context must also be distinguished from eating-disorder psychopathology while recognizing that the two can coexist. Food insecurity is an underrecognized contributor to binge eating and other disordered eating patterns, and periods of involuntary restriction may produce behaviors that are easily misunderstood when clinicians do not assess access to food, financial instability, and household conditions (Abene et al., 2023). A culturally responsive assessment therefore avoids both errors: pathologizing adaptive responses to scarcity and overlooking eating-disorder symptoms because food insecurity is present.

The proposed framework hypothesizes that embodied and reflective training may support the processes relevant to early detection by influencing what clinicians are prepared to notice, question, and explore further. This proposed connection has not yet been established empirically; existing literature supports the component processes—such as bias awareness, clinician self-awareness, and the need for improved eating disorder preparation—rather than a direct effect of embodied pedagogy on detection outcomes. The goal is not to make every counselor an eating-disorder specialist. It is to prepare all mental health clinicians to recognize possible concerns, ask informed and non-stigmatizing questions, avoid using appearance as a proxy for severity, assess sociocultural context, and refer promptly for specialized medical, nutritional, and psychological evaluation when indicated. Early intervention research supports the importance of targeted prevention, accessible screening, and intervention before symptoms become more severe or entrenched (Koreshe et al., 2023). In this conceptual model, body-related self-awareness is proposed as one means through which clinicians may become more able to recognize beliefs, biases, and avoidant responses that could influence whether eating-disorder concerns enter the clinical frame.

## 6. A Four-Domain Framework for Embodied and Reflective Body-Based Learning

This article proposes a four-domain framework for preparing mental health trainees to recognize and respond to eating disorder and body image concerns: foundational knowledge, reflective body-related self-awareness, embodied and creative practice, and clinical translation. The domains are interconnected rather than strictly sequential. Knowledge informs reflection, reflection deepens embodied awareness, and all three must ultimately influence assessment, consultation, referral, and intervention.

The present framework is intended to complement rather than replace Irvine and Labarta’s (2024) 3 Cs and existing counselor education expectations. Domains 1, 2, and 4 intentionally overlap with established priorities: foundational knowledge supports informed and culturally responsive eating disorder recognition; reflective body-related self-awareness applies existing expectations for counselor self-awareness specifically to beliefs and assumptions about food, weight, health, and bodies; and clinical translation reinforces assessment, consultation, referral, and interdisciplinary collaboration. The framework’s primary differential contribution is Domain 3, embodied and creative practice. Whereas existing models emphasize what trainees should know, reflect upon, and consider within sociocultural and interdisciplinary contexts, Domain 3 proposes structured opportunities for trainees to notice and examine moment-to-moment bodily and relational responses during simulated or clinical encounters and to process those responses through reflective, creative, and supervisory practices. For example, a trainee may move beyond cognitively identifying weight bias to noticing tension, urgency, avoidance, comparison, or an impulse to reassure during a body-related clinical interaction, then examine that response through reflection and supervision before returning to client-centered assessment. Thus, the framework does not propose four wholly novel competencies; rather, it organizes established training priorities around a distinct embodied pedagogical component and connects that component to supervised clinical practice.

The domains may be infused into existing courses, including diagnosis, assessment, multicultural counseling, human development, practicum, and internship, or organized into a dedicated eating disorder and body image training module. The framework does not presume that every trainee will become an eating disorder specialist. Rather, it aims to prepare mental health professionals to recognize possible concerns, respond without stigma, understand the limits of their competence, and refer appropriately.

## 7. Domain 1: Foundational Knowledge

Trainees need accurate and current knowledge about feeding and eating disorder diagnoses, warning signs, medical and nutritional risk, screening and assessment, levels of care, body image development, weight stigma, prevention, and multidisciplinary treatment. Foundational knowledge protects against the assumption that personal experience, general counseling skill, or familiarity with body image language is sufficient preparation for identifying eating disorder concerns.

At minimum, trainees should be able to recognize major eating disorder presentations, including those that do not conform to stereotypical assumptions about body size, gender, race, age, or socioeconomic status. They should understand that eating disorder severity cannot be determined by appearance alone and that clients in larger bodies may experience clinically significant restriction, malnutrition, compensatory behavior, and medical instability. Trainees also need familiarity with common psychological, behavioral, medical, and nutritional warning signs and with the roles of physicians, registered dietitians, psychiatrists, and other members of an interdisciplinary treatment team.

This knowledge should influence what trainees are prepared to notice and ask. Clients may not initially identify their experiences as disordered eating. They may instead present with anxiety, depression, perfectionism, gastrointestinal complaints, fatigue, compulsive exercise, body shame, rigid food practices, or distress related to health and appearance. A clinician who understands these possible presentations is less likely to dismiss symptoms because the client does not resemble a familiar eating disorder stereotype.

Foundational preparation is especially important given persistent training gaps. Labarta et al. (2023) found that many clinicians received little or no graduate-level eating disorder education and identified limited training as a major challenge in treatment. Foundational instruction therefore should be intentional rather than dependent on a single lecture, optional elective, or incidental exposure during practicum.

## 8. Domain 2: Reflective Body-Related Self-Awareness

Knowledge alone does not address how clinicians’ personal histories and cultural assumptions affect clinical perception. Trainees also need structured opportunities to examine their own relationships with food, weight, health, exercise, appearance, ability, sexuality, aging, illness, and bodily vulnerability. This capacity may be described as body-related self-awareness: the clinician’s ability to identify and examine personal beliefs, emotions, values, biases, and narratives related to bodies and to consider how these may influence care.

Reflection may include family rules about food, cultural beauty ideals, spiritual meanings assigned to the body, beliefs about exercise and discipline, experiences of weight stigma, health anxiety, disability, medical vulnerability, or body-based shame and pride. The purpose is not autobiographical disclosure for its own sake. Rather, the goal is to help trainees identify how these experiences may shape what they notice, normalize, praise, avoid, or pathologize.

Body-related self-awareness also requires examining implicit assumptions. A trainee may associate thinness with health, weight loss with success, exercise with virtue, or larger body size with noncompliance. Another may assume that restrictive eating is concerning only when a client appears visibly underweight. Still another may avoid asking about weight, food, or compensatory behaviors because these topics feel intrusive or shame-inducing. Such reactions are not evidence that a trainee is unfit. They are clinically relevant material that must become available for reflection and supervision.

The goal is not to eliminate every automatic response. That expectation would be unrealistic and could encourage defensiveness. The goal is awareness, humility, and choice. A clinician who notices a reaction can question it, seek supervision, and choose a more deliberate response. A clinician who remains unaware of the reaction is more likely to enact it through silence, reassurance, judgment, avoidance, or premature diagnostic conclusions.

Reflective activities should remain clinically focused. Trainees might consider what they assume a client’s body communicates about health, motivation, self-control, attractiveness, or risk; which body-related topics they find easiest or most difficult to discuss; and how their histories might lead them to overidentify, reassure too quickly, become overly directive, or avoid necessary assessment.

## 9. Domain 3: Embodied and Creative Practice

Reflective awareness is often approached cognitively, yet clinicians also respond to clients through sensation, emotion, posture, movement, and physiological activation. Embodied awareness refers to the clinician’s moment-to-moment capacity to notice bodily cues such as tension, numbness, constriction, urgency, discomfort, or withdrawal within themselves and within the therapeutic interaction. Rodríguez and Wilkinson (2026) describe embodied self-awareness as an often-neglected dimension of counselor preparation that supports self-regulation, therapeutic presence, countertransference awareness, and relational attunement.

Embodied and creative practices help trainees move from thinking about the body to noticing how bodily experience unfolds during clinical work. During role-play, trainees might observe changes in breathing, posture, tension, or the urge to reassure, redirect, compare, advise, or withdraw when food, weight, or body image concerns emerge. Grounding practices can help trainees remain present while asking direct questions that may evoke discomfort. Somatic countertransference tracking can help them distinguish between information arising from the client interaction and reactions rooted in their own experiences or cultural conditioning.

Creative approaches may include body mapping, visual timelines, reflective writing, collage, metaphor work, or mapping the connections among body-related thoughts, emotions, sensations, and behaviors. These activities may help externalize narratives that are difficult to access through discussion alone. A trainee might visually represent messages received about hunger, health, attractiveness, masculinity, femininity, disability, strength, or control and then consider how those messages may affect clinical assumptions.

These exercises should always be connected to professional application. The central question is not simply, “What did I feel?” but “How might what I noticed influence my assessment, language, relational presence, or treatment decisions?” Embodied reactions should not be interpreted as objective truths about clients. They should be treated as possible sources of information that require curiosity, context, and supervision.

Ethical safeguards are necessary. Faculty should not require students to disclose personal eating disorder histories, trauma, weight, medical diagnoses, or other sensitive experiences. Students should have choices regarding the depth of disclosure and the form of participation. Activities should avoid mandatory weighing, calorie tracking, public body comparison, appearance evaluation, or other practices that could reproduce eating disorder culture or evoke unnecessary harm.

## 10. Potential Harms, Limits, and Supervisory Safeguards

Increased attention to clinicians’ embodied responses carries potential risks as well as potential benefits. A clinician who gives excessive weight to an internal sensation or emotional response could increase false-positive impressions, project personal body or eating histories onto a client, or pathologize culturally normative eating and body-related practices. Accordingly, embodied responses should not be conceptualized as validated diagnostic indicators or as direct evidence about a client. Somatic or emotional countertransference is better understood within this framework as material for reflective inquiry: a response that may warrant curiosity about the clinician, the therapeutic relationship, or the clinical situation but that requires corroboration before influencing assessment or referral. The empirical literature on countertransference supports its relevance to clinical reflection, but the meaning of any individual therapist response cannot be assumed from the response alone.

Supervision is therefore essential for distinguishing potentially useful awareness from projection, bias, or unrelated clinician activation. Trainees can be encouraged to examine several questions before assigning clinical meaning to an embodied response: What observable client information prompted concern? What alternative explanations are possible? Could the reaction reflect the trainee’s own history, current emotional or physiological state, cultural learning, weight bias, or assumptions about health? What additional client-centered assessment is needed before drawing a conclusion? Supervisors should redirect trainees from internal reaction back to independent clinical information, including the client’s reported experience, behavioral and contextual indicators, appropriate screening, and consultation when indicated. When an embodied response is not supported by additional clinical evidence, it should remain information about the clinician’s experience rather than becoming a conclusion about the client.

This distinction is particularly important in culturally responsive eating disorder assessment. Restriction, fasting, eating patterns, body change, food practices, and exercise may have meanings related to religion, culture, food access, medical conditions, disability, athletic participation, or family practices that cannot be inferred from the behavior alone. Embodied discomfort or unfamiliarity on the part of the clinician should therefore increase curiosity rather than increase diagnostic certainty. The purpose of embodied self-awareness is not to lower the evidentiary standard for identifying pathology, but to help clinicians recognize when their own reactions might otherwise lead them either to prematurely dismiss a concern or to overinterpret one.

Experiential training itself also warrants caution. For counselor education, safeguards should therefore include advance notice that coursework contains experiential body- and eating-related material; meaningful options to decline or modify activities without academic penalty; equivalent alternative assignments; prohibition of required disclosure of eating disorder history, weight, body measurements, caloric intake, or other sensitive personal information; and clear referral pathways when course material evokes significant distress. Instructors should also possess sufficient competence in eating disorders, body image, and experiential facilitation to recognize when an exercise is becoming activating or clinically inappropriate. Evaluation should focus on students’ professional reflection and clinical application rather than the content of their personal body histories. These safeguards preserve the educational value of embodied learning while recognizing the power differential inherent in asking students to engage personally with body-related material in an evaluative setting.

## 11. Domain 4: Clinical Translation

Embodied reflection becomes professionally meaningful when it changes clinical behavior. Trainees should practice broaching eating and body-related concerns, asking direct but nonjudgmental questions, recognizing possible urgency, consulting with other professionals, and making timely referrals. This domain transforms internal awareness into ethical clinical action.

Students should learn to assess eating patterns, restriction, binge eating, loss-of-control eating, purging, exercise rigidity, compensatory movement, food rules, fear foods, body checking, body avoidance, weight history, medical symptoms, shame, secrecy, and functional impairment. They should also consider food access, cultural food practices, religious fasting, medication-related body changes, disability, and financial conditions. These contextual factors may shape eating behavior and must be approached without assuming that all restriction, body change, or food-related distress carries the same meaning.

Clinical translation also involves learning to maintain eating disorder and body image concerns within the differential diagnosis when available information warrants further inquiry. This does not mean assigning a provisional diagnosis or assuming that ambiguous symptoms indicate an eating disorder. Rather, it means resisting premature exclusion long enough to ask informed questions, gather relevant contextual information, use appropriate screening when indicated, consult when necessary, and determine whether additional medical, nutritional, psychiatric, or specialized eating disorder evaluation is warranted. This approach is intended to raise the clinician’s index of suspicion when relevant indicators are present while preserving careful differential assessment and avoiding over-pathologizing. Countertransference awareness is central to this domain. Trainees should be able to identify how their histories or assumptions could lead them to praise weight loss without understanding its cause, minimize compulsive exercise, collude with moralized health beliefs, avoid discussing eating behavior, overidentify with a client, or become overly directive. Labarta et al. (2023) emphasized that the personal nature of food, eating, and body image may contribute to countertransference and recommended reflection on weight stigma, diet culture, triggers, and personal beliefs as part of counselor preparation.

Clinical translation should be observable. Students can demonstrate competence through role-plays, case simulations, documentation exercises, consultation decisions, and referral planning. Evaluation may consider whether the trainee asks direct and respectful questions, avoids appearance-based assumptions, recognizes possible medical urgency, distinguishes personal reactions from client information, identifies the limits of personal competence, and develops an appropriate interdisciplinary response.

## 12. Integrating the Four Domains

The four domains function together. Foundational knowledge without reflection may leave body-related biases unchanged. Reflection without knowledge may produce insight without sufficient diagnostic or referral competence. Embodied practice without clinical translation may increase awareness without changing client care. Clinical skills without body-related self-awareness may reproduce stigma, avoidance, or culturally narrow assumptions.

Whereas prior counselor education scholarship has identified the need for stronger eating disorder preparation, this framework operationalizes that need through a developmental pathway from knowledge to reflection, embodiment, and clinical action. Its purpose is not only to help trainees understand eating disorders, but also to help them recognize how personal history, cultural values, bodily responses, and clinical behavior interact when eating and body-related concerns enter, or fail to enter, the therapeutic frame.

## 13. Applied Course Example: From Syllabus to Training Model

The following course example is offered as an illustration of how the four-domain framework may be translated into graduate counselor education rather than as an empirically evaluated intervention. A layout of the course is shown in Table 1. The seven-session graduate-level eating disorders course was developed to integrate a numerous competency within the counseling field (Taylor, 2024). The course has been delivered across 7 cohorts of graduate counseling students. Although no formal outcome evaluation has been conducted, implementation has provided preliminary observations regarding the feasibility and acceptability of incorporating reflective and experiential body-related learning within a short graduate course. Students have reported an increased awareness of their own body image concerns and how this affects their understanding of client’s body image issues. Cohorts have also discussed an increased confidence in being able to identify disordered eating when it arises in the counseling office. These observations should be understood as informal implementation experience rather than evidence of effectiveness. Rather than presenting eating disorders as an isolated diagnostic category, the course examines how eating, body image, health, and embodiment intersect with trainees’ developing clinical identities. The course was developed within a U.S.-based, faith-integrated counselor education context, which informed its inclusion of spiritual and religious dimensions of body image, eating, health, and clinical identity. These elements are not proposed as universal components of the framework. In secular programs, or in cultural and international contexts in which spiritual integration is approached differently, the underlying reflective activities could instead examine locally relevant cultural, philosophical, familial, or meaning-making influences on embodiment. Accordingly, the course is intended as an adaptable example rather than a prescriptive curriculum.

The course begins with foundational instruction in DSM-5-TR feeding and eating disorder diagnoses, medical and nutritional risk, body image development, and multidisciplinary care. Students also complete self-assessments related to eating attitudes, body image, intuitive eating, and family food rules. These assessments are graded on a complete/incomplete basis, and students are not evaluated on their personal responses. This safeguard promotes reflection without requiring disclosure of sensitive information.

Students then move from self-observation to clinical application. They create a word cloud representing language and beliefs associated with food, body image, and appearance and later revisit these themes through cognitive restructuring and a body image collage. Additional activities examine positive and negative body image and the influence of developmental, biological, cultural, spiritual, relational, and societal factors. Together, these assignments help students identify their own body-related filters while considering how similar influences may shape client experience.

The course culminates in a final critique and reflection paper that integrates eating disorder knowledge, body image scholarship, multicultural and spiritual considerations, countertransference, and an original treatment or prevention idea. Students also develop a community referral guide that includes physicians, registered dietitians, psychiatrists, obstetrician-gynecologists, hospitals, and higher levels of eating disorder care. This assignment moves students from personal insight to professional responsibility by requiring them to identify concrete resources for clients whose needs exceed the counselor’s scope of competence.

## 14. Policy Recommendations for Training Programs

If body-related self-awareness contributes to ethical assessment and early detection, it should not remain an optional specialty topic limited to eating disorder coursework. Mental health training programs can integrate embodied and reflective learning into professional identity development, multicultural counseling, ethics, assessment, supervision, and clinical practice. CACREP-accredited programs already assess student knowledge, skills, professional dispositions, and curricular effectiveness; therefore, eating disorder detection and body-related self-awareness can be incorporated within existing educational structures rather than treated as an entirely separate training requirement (Council for Accreditation of Counseling and Related Educational Programs [CACREP], 2024).

A freestanding course focused on eating disorders offers one pathway for developing these competencies, particularly when programs have the curricular flexibility, faculty expertise, and institutional resources to support specialized instruction. However, requiring an additional course may not be feasible for many graduate counseling programs given existing credit-hour requirements and the breadth of clinical competencies students are expected to develop. Further, the purpose of this framework is not to suggest that eating disorders should be prioritized over other significant clinical concerns, such as substance use disorders, suicide prevention, trauma, or other areas requiring specialized knowledge. Rather, eating disorders provide a particularly important example of a clinical concern that may be overlooked when counselors rely on stereotypical presentations, fail to examine their own assumptions, or do not attend to embodied information arising within the counseling relationship. Thus, the proposed competencies can be infused throughout existing coursework without requiring programs to displace other essential content.

For example, multicultural counseling courses could invite students to examine how cultural ideals regarding weight, health, attractiveness, gender, race, socioeconomic status, disability, and athletic identity influence assumptions about who is perceived as having an eating disorder. Students might complete a structured reflection identifying messages they have received about food, bodies, weight, and health and consider how these beliefs could influence clinical assessment. Such an activity would connect body-related self-awareness directly to multicultural humility and the recognition of counselor bias.

Within assessment and diagnosis courses, instructors could incorporate eating disorder screening into existing case conceptualization exercises. Rather than presenting eating disorders only through prototypical cases involving an underweight young woman explicitly reporting food restriction, students could encounter clients presenting primarily with anxiety, depression, trauma symptoms, gastrointestinal complaints, excessive exercise, food insecurity, or concerns about body image. Students could then be asked to identify additional assessment questions, determine whether further screening is indicated, and reflect on what characteristics of the client initially increased or decreased their suspicion of an eating disorder. This approach teaches both diagnostic knowledge and awareness of the assumptions that shape clinical decision making.

Ethics and professional orientation courses provide another opportunity for integration. Case discussions could examine the ethical consequences of missed assessment, weight-based assumptions, practicing outside one’s scope of competence, or failing to refer when eating disorder symptoms or medical instability exceed a counselor’s training. Students could also consider how personal beliefs about weight, dieting, exercise, or health may influence clinical recommendations even when those beliefs are experienced as normative or well intentioned. In this context, embodied self-awareness becomes part of ethical self-monitoring rather than an isolated wellness exercise.

The most direct opportunity for application may occur during practicum and internship, when students are simultaneously developing clinical judgment and becoming more aware of their own internal responses to clients. Supervisors could periodically invite trainees to attend not only to what they are thinking about a client but also to what they notice within themselves during sessions involving food, weight, appearance, control, exercise, or body-related distress. For example, a student might notice an impulse to reassure a client about appearance, avoid asking about eating behaviors, assume that a higher-weight client could not be restricting intake, or experience discomfort when discussing weight directly. Supervision can help the trainee examine these reactions without treating them as evidence about the client. The goal is to develop the capacity to notice embodied and emotional responses, consider whether bias or countertransference may be present, and then return to systematic assessment and client-centered inquiry.

A program could therefore operationalize the four-domain framework longitudinally rather than locating it within a single course. Foundational knowledge might first be introduced in psychopathology or diagnosis; reflective body self-awareness could be developed through multicultural and professional identity coursework; embodied and creative practice could be introduced through skills courses and experiential exercises; and clinical translation could occur during practicum, internship, and supervision. Faculty could then evaluate students’ development through existing case conceptualizations, reflective assignments, skills evaluations, supervision documentation, and dispositional assessment processes. In this model, no single course carries responsibility for eating disorder competency. Instead, students encounter progressively more complex opportunities to recognize symptoms, examine assumptions, tolerate body-related clinical material, and translate awareness into responsible assessment and referral.

This cross-curricular approach may also be more sustainable than relying solely on specialized electives because it positions eating disorder detection within broader counselor competencies that programs are already responsible for developing. The underlying skills (recognizing non-stereotypical presentations, examining implicit assumptions, attending to countertransference, tolerating clinical discomfort, conducting appropriate screening, and knowing when to seek consultation or referral) extend beyond eating disorders. Embedding these practices across counselor education therefore strengthens not only eating disorder recognition but also the broader development of reflective, culturally responsive, and ethically attentive clinicians.

## 15. Recommendations

1. Name body-related self-awareness as part of professional self-awareness. Programs should explicitly address how beliefs about food, body size, exercise, appearance, health, ability, and embodiment may influence clinical perception and decision-making.

2. Infuse body-related learning across the curriculum. Relevant content can be incorporated into multicultural counseling, ethics, assessment, diagnosis, lifespan development, trauma, psychopathology, practicum, internship, and supervision without requiring additional credit hours. Rather than adding separate units, faculty can integrate eating disorder and body-related material into assignments and learning activities that already address multicultural awareness, diagnostic assessment, ethics, case conceptualization, and clinical self-reflection. For example, an existing assessment assignment case could include subtle eating disorder indicators, a multicultural reflection could examine weight stigma and cultural body ideals, and practicum or internship supervision could invite trainees to notice and reflect on embodied reactions that arise during client work. In this way, eating disorder and body-related awareness function as applications of competencies already required within counselor preparation rather than as additional content that must displace other essential topics. Specifically in practicum and internship, if a student reports having a client with an eating disorder, the client reflecting upon their own body image history and current experience could be applied.

3. Require basic eating disorder detection and referral preparation. All trainees should learn to ask direct and respectful questions about eating disorder symptoms, recognize possible medical and nutritional risk, understand the limits of their competence, and refer to multidisciplinary professionals when indicated.

4. Address weight stigma and health moralism. Programs should help trainees examine cultural messages that equate weight, food choices, fitness, or appearance with morality, discipline, worth, or health.

5. Include body-related countertransference in supervision. Supervisors should invite reflection on embodied reactions, discomfort, avoidance, judgment, urgency, and overidentification when body-related concerns emerge in clinical work.

6. Assess clinical competence rather than private disclosure. Programs can evaluate students’ assessment skills, case conceptualization, recognition of bias, use of supervision, and referral planning without grading personal body histories or requiring disclosure of sensitive experiences.

## 16. Future Research

The proposed relationship between embodied and reflective pedagogy and eating disorder recognition remains an empirical question. Future research should therefore examine whether this approach influences clinical behavior rather than assuming that increased self-awareness or confidence translates into more accurate assessment. Studies might examine performance in standardized clinical encounters, appropriateness of screening and referral decisions, sensitivity to nonstereotypical presentations, and, where feasible, the time between clinically relevant indicators and consultation or specialist referral.

Research designs should also account for important methodological limitations. Students who elect an eating disorder course may differ from other trainees in prior knowledge, personal experience, professional interests, or motivation, making self-selection a potential confound. Self-report measures of confidence, awareness, or avoidance may also be vulnerable to demand characteristics and socially desirable responding, particularly when students understand the goals of the training. Future studies should therefore incorporate behavioral outcomes and, when possible, independent or blinded evaluators rather than relying primarily on instructor-scored reflection. Preregistration of primary hypotheses, outcomes, and analytic decisions would further strengthen this work.

Research should also examine unintended outcomes. Greater attention to embodied reactions should not be assumed to improve clinical judgment and could theoretically contribute to false-positive impressions, projection, or inappropriate pathologizing if internal responses are overinterpreted. Future studies should therefore evaluate both appropriate recognition and referral and inappropriate escalation of concern. Such work would help determine whether, for whom, and under what training conditions embodied and reflective pedagogy contributes to clinically useful eating disorder preparation.

## 17. Discussion: From Awareness to Action

Mental health education has made significant progress in emphasizing cultural humility, ethical practice, professional values, reflective development, and the clinical use of self. The next step is to make the body explicit within that formation. The clinician’s body is not irrelevant to clinical work. It is part of how clinicians notice, avoid, regulate, empathize, interpret, and respond. When the clinician’s relationship with the body remains unexamined, body-related bias and avoidance may shape care without being named.

The proposed framework invites programs to move beyond awareness as a vague ideal and toward structured educational design. Students need knowledge, but they also need reflective and embodied practice. They need assignments that help them notice their own body narratives, language, and assumptions. They need supervision that makes body-related countertransference discussable. They need practice asking difficult but necessary questions. They need training that understands eating disorder detection as both a diagnostic skill and a relational skill.

This approach also broadens the relevance of eating disorder education. Eating disorder training is not only for students who intend to specialize. It is a vehicle for teaching clinical self-awareness, multicultural humility, medical collaboration, bias recognition, and embodied therapeutic presence. In this sense, the eating disorder classroom can become a site of broader professional formation.

## 18. Conclusions

Mental health training programs routinely teach that the clinician’s self matters. This article proposes that the clinician’s relationship with the body warrants similar attention. Body-related self-awareness may represent an important dimension of ethical, culturally responsive, and clinically attentive practice, particularly when clinicians encounter concerns involving food, weight, health, appearance, and embodiment.

The literature reviewed in this article supports several components of this argument while also establishing important boundaries around its claims. Existing evidence demonstrates that weight bias can influence clinical judgment, that graduate preparation in eating disorders remains limited, and that embodied awareness may support therapeutic presence, self-regulation, and countertransference awareness. What has not yet been established is whether teaching embodied and reflective practices improves eating disorder detection itself. The proposed four-domain framework therefore represents a conceptual and pedagogical hypothesis rather than an empirically validated pathway to earlier recognition.

The value of the framework lies in making that hypothesis testable. Future research should examine whether trainees who receive structured embodied and reflective preparation demonstrate more accurate assessment, fewer stereotype-based omissions, appropriate use of further screening and consultation, and more timely referral without increasing false-positive identification or culturally inappropriate pathologizing. Until such outcomes are established, embodied responses should be understood as material for reflection and supervision rather than diagnostic evidence.

The next step, therefore, is not to assume that greater embodied awareness produces better detection, but to determine whether and under what conditions it contributes to more thoughtful, culturally responsive, and clinically appropriate inquiry.

## Figures and Tables

**Table 1:** Course-Based Translation of Embodied and Reflective Training.

Course Element	Embodied/Reflective Function	Clinical Training Purpose
Pre/post self-assessment	Invites students to notice body image, eating, intuitive eating, and family-rule patterns over time.	Supports self-awareness without grading personal disclosures.
Cognitive word cloud	Externalizes body-related language and cognitions.	Helps students notice the words and assumptions they may bring into clinical work.
Holistic body image reflection	Connects body image to cultural, spiritual, biological, relational, and developmental sources.	Strengthens multicultural case conceptualization.
Cognitive restructuring/body collage	Practices reframing, dialectical thinking, and creative engagement with body narratives.	Provides an adaptable intervention idea for clients.
Countertransference section	Links personal body story to possible clinical reactions.	Normalizes supervision of body-related bias, avoidance, and overidentification.
Referral/resource list	Moves awareness into systems-level action.	Builds competence in multidisciplinary care and early referral.

## Data Availability

No new data were created or analyzed in this study. Data sharing is not applicable to this article.
